# Supplementary material for: Genome-wide association analysis of Russian wheat aphid (Diuraphis noxia) resistance in Dn4 derived wheat lines evaluated in South Africa
Source: PLoS One. 2020 Dec 28;15(12):e0244455. doi: 10.1371/journal.pone.0244455 (PMC7769470; doi:10.1371/journal.pone.0244455)
Supplement: Attachment — Submitted filename: Response to Reviewers.docx [file pone.0244455.s006.docx]

Academic Editor

PLOSONE

2 November 2020

Submission of a revised manuscript ID: PONE-D-20-27285

Dear Dr Aimin Zhang

Thank you very much for your positive comments on our manuscript ID PONE-D-20-27285 titled “Genome-wide association analysis of Russian wheat aphid (*Diuraphis noxia*) resistance in *Dn4* derived wheat lines evaluated in South Africa” which we have revised along the reviewers’ comments.

Please find below point-by-point response on how we integrated each of the comments into the new draft. A version of the manuscript showing the editions made from the initial draft is also provided with tracked changes.

With best regards

Lavinia Kisten

Corresponding author

**Reviewer 1**

***Comment***

*At the end of the discussion, the authors may wish to consider adding plant level examples of the effect of temperature and light on expression of resistance i.e. Hessian fly resistance gene sensitivity to temperature (Chen et al. 2014. J. Econ. Entomol. 107:1266) and wheat stem sawfly resistance gene sensitivity to light intensity (Varella et al. 2016 Plant Breed.135: 546).*

**Response**

The additional examples of the effect temperature and light have on gene expression have been added to the discussion. **Page 34 Lines 3-7.**

***Comment***

*Minor grammar edits were included in the reviewer attachment.*

**Response**

The grammatically errors were corrected. **Throughout document**

**Reviewer 2**

***Comment***

*Representative samples are required for GWAS analysis. Only 32 samples are used in this paper. The author should elaborate on why these samples are used and how these samples can obtain accurate results of correlation analysis.*

**Response**

The GWAS was based on 181 sampled individuals. These individuals were obtained from 32 genotypes. Within a genotype, there were individuals that exhibited extreme and opposite reactions to a particular RWA biotype. Due to the differential reactions among individuals of the same genotype, it was important to identify the individuals rather than to bulk them as single genotypes. The differences among individuals of same genotypes could arise due to subtle mutations in individuals and crossing over (or recombination) between chromatids of homologous chromosomes during meiosis, which are then inherited by different individual kernels of the same spike. These causes are well known in breeding of self-pollinating species such as wheat where single seed descent is used to counter such variation from influencing breeding progress. Thus, the 181 individuals were deemed sufficient to conduct a GWAS despite their development from 32 genotypes. **Page 8 Lines 185-198.**

***Comment***

*The relationship between the 32 phenotyped samples and the 181 genotyped samples was not clearly described.*

**Response**

The test individuals were developed based on the response of the thirty-two genotypes to four RWA biotypes. Each genotype was planted and inoculated with four different RWA biotypes in three replicates of 5 plants each. After inoculation and disease severity scoring, plants of the same genotype in the same replicate that exhibited differential response to RWA were bulked separately. A genotype could potentially have individual plants that exhibited susceptibility, intermediate and resistance to RWA. The individuals in the intermediate reaction grouped were not sampled. Only individuals with extreme ie either susceptible or resistant reaction were sampled and bulked accordingly per biotype. Some genotypes consisted of individuals with similar reaction to a particular RWA biotype, in which case a single bulk sampled would be collected. Other genotypes consisted of individuals with differential reaction and at most, two bulk samples of susceptible and resistant samples would be collected per biotype. In total, 181 individuals with differential reaction to the four RWA biotypes were sampled for genotyping and used for downstream analyses. **Page 8 Lines 185-198.**

***Comment***

*Fig. 3A shows that LD has two peaks. Generally, LD is gradually attenuated, what is the reason for forming the double peak of LD?*

**Response**

The double peaks in the LD plot could have been caused by the high LD values found among markers on the two LD blocks, one block on chromosome 1 and the other on chromosome 6. The first peak of R2 values above 0.6 was found for markers occurring within the 0-20 Mbp range on chromosome 1 and the second peak occurred within a 30 Mbp range from 280-310 Mbp distance on chromosome 6. Although LD plots would be expected to have a single peak that attenuates with over genetic distance, instances of multiple peaks do occur in structured populations characterised by different haplotypes. Joukhadar et al 2013 found that the double peaks on the LD plot were smoothed by the removal of markers in distant LD blocks that exhibited high R2 values on different chromosomes. Thus, the double peaks in LD found in this study could be attributed to the structure of the germplasm, which was delineated into winter and spring wheat clusters. Each cluster consisted of genotypes with variable reaction to the different aphid biotypes. **Page 22 Lines 9-12; Page 31 Lines 18-25; Page 32 Lines 1-5.**

***Comment***

66

**Response**

It is unclear to what this comment is referring to, therefore it was not addressed.

***Comment***

*Page 16, line 1 an average.*

**Response**

The error has been corrected. **Page 20 Line 9.**

***Comment***

*Page 5, line 145-147 too long sentence. please split it.*

**Response**

The sentence has been simplified. **Page 7 Line 150.**

***Comment***

*Fig. 1 and Fig. 4 is too blurry to be clearly distinguished, please give a higher resolution image.*

**Response**

The quality of the figures has been improved and higher resolution images were submitted.

***Comment***

*Fig. 21 line 8, ti should be to*

**Response**

The error has been corrected. **Page 26 Line 8.**
