# Supplementary material for: Genome-wide association analysis of Russian wheat aphid (Diuraphis noxia) resistance in Dn4 derived wheat lines evaluated in South Africa
Source: PLoS One. 2020 Dec 28;15(12):e0244455. doi: 10.1371/journal.pone.0244455 (PMC7769470; doi:10.1371/journal.pone.0244455)
Supplement: Attachment — Submitted filename: Response to Reviewers.docx [file pone.0244455.s007.docx]

Academic Editor

PLOSONE

2 December 2020

Submission of a revised manuscript ID: PONE-D-20-27285

Dear Dr Aimin Zhang

Thank you very much for your positive comments on our manuscript ID PONE-D-20-27285 titled “Genome-wide association analysis of Russian wheat aphid (*Diuraphis noxia*) resistance in *Dn4* derived wheat lines evaluated in South Africa” which we have revised along the reviewers’ comments.

Please find below point-by-point response on how we integrated each of the comments into the new draft. A version of the manuscript showing the editions made from the initial draft is also provided with tracked changes.

With best regards

Lavinia Kisten

Corresponding author

**Reviewer 3**

***Comment***

*In page 6, line 137-139, Table 1 was stated that there are 25 genotypes containing Dn4 genes. However, there are 24 genotypes carrying the Dn4 gene in table 1. Also, the sum of the specified numbers of genotypes makes 33 in the same lines.*

**Response**

The error has been corrected. **Page 6 Line 137.**

***Comment***

*In page 14, line 4-5, the sentence*

*“Three of the MTRWA92 breeding lines (91, 120 and 160), contained the 195 bp fragment, while the remaining nine lines (93, 114, 115, 121, 145, 149, 150, 155, 158) had the 175 bp fragment.”*

*MTRWA92-160 line does not contain the 195 bp fragment in Table 2.*

**Response**

The error has been corrected within the paragraph. **Page 14 Lines 4-5.**

***Comment***

*In page 14, line 11-12, the sentence*

*“The 125 bp fragment was amplified in PI372129, Halt, 12 BondCL, Yumar and all of the 18 FAWWON-SA 64 plants.”*

*Also, Corwa genotype contains the 125 bp fragment according to Table2.*

**Response**

Corwa has been added to the list of genotypes that contain the 125 bp fragment. **Page 14 Line 12.**

***Comment***

*In page 18, line 10-11,*

*“MTRWA92-161 was resistant to RWASA1, RWASA3 and RWASA4 and showed a moderate resistance to RWASA2.”*

*This information was given two sentences before in the same paragraph, in that;*

*“Genotypes MTRWA92- 161 and MTRWA92-93 were moderately resistant to RWASA2 and showed a high level of resistance to RWASA1, RWASA3 and RWASA4*

**Response**

The duplicated description of the result has been removed **Page 18 Line 10.**

**Reviewer 4**

***Comment***

*It is suggested that some description in the discussion for the resistance level to of the test wheat lines to the other wheat aphids, because the several wheat aphids species usually concur at the same.*

**Response**

The resistance response of the genotypes to other aphid species was not assessed in this study. Our focus was on the Russian aphid, although the other species of aphids may occur simultaneously with the Russian aphid. The reaction of the genotypes to other aphid species could not be immediately established from literature. However, reaction of two of the genotypes, namely Hatcher and ThunderCL, to other insect pests, namely Hessian fly and green bug were included in the discussion as follows:

“Furthermore, broad spectrum resistance to multiple insect pests is desired in cultivars and breeding lines as multiple pests often occur simultaneously in the field. ThunderCL exhibited a high level of resistance to RWASA1, 2 and4 while Hatcher displayed moderate resistance to RWASA1 and RWASA2. Additionally, these cultivars are reportedly resistant to the Hessian fly (*Mayetiola destructor* Say), whereas they are susceptible to green bug (*Schizaphis graminum* Rondani) [62,63].” **Page 28 Lines 18-23.**
